# Gradient diffusion antibiogram used directly on bronchial aspirates for a rapid diagnosis of ventilator-associated pneumonia

**DOI:** 10.1186/s13756-019-0640-1

**Published:** 2019-11-14

**Authors:** Almudena Burillo, Viviana de Egea, Raffaella Onori, Pablo Martín-Rabadán, Emilia Cercenado, Laura Jiménez-Navarro, Patricia Muñoz, Emilio Bouza

**Affiliations:** 1 Department of Clinical Microbiology and Infectious Diseases, Hospital General Universitario Gregorio Marañón, Doctor Esquerdo 46, 28007 Madrid, Spain; 2 Department of Medicine, School of Medicine, Universidad Complutense de Madrid, Ciudad Universitaria, 28040 Madrid, Spain; 3 Instituto de Investigación Sanitaria, Hospital Gregorio Marañón, Doctor Esquerdo 46, 28007 Madrid, Spain; 4 CIBER Enfermedades Respiratorias-CIBERES (CB06/06/0058), Servicio Madrileño de Salud, Doctor Esquerdo 46, 28007 Madrid, Spain

**Keywords:** Antibiogram, Ceftobiprole, Ceftolozane-tazobactam, Microbial sensitivity tests, Rapid diagnosis, Respiratory tract samples

## Abstract

**Background:**

In patients with suspected ventilator-associated pneumonia, a rapid etiological diagnosis is crucial as incorrect or delayed treatment in the first few hours leads to a worse prognosis and a higher mortality rate. This study examines the efficacy of a rapid antibiogram on bronchial aspirates in patients with suspected ventilator-associated pneumonia (VAP).

**Methods:**

The direct gradient diffusion susceptibility testing method (GDM) on respiratory samples was compared with a standard broth microdilution method (BMD) after quantitative cultures in patients with suspicion of VAP. Samples were preselected by Gram staining (for good quality microbiological samples with a predominant single bacterial morphotype). The antibiotics tested were ceftazidime, ceftobiprole, ceftolozane-tazobactam, meropenem, doripenem, and tedizolid.

**Results:**

Over a 16-month study period, 445 bronchial aspirate samples were selected from 1376 samples received at our laboratory from 672 adult patients. By direct plating on Mueller-Hinton agar, we recovered 504 (95.5%) of the 528 microorganisms identified by the standard semiquantitative method. Antimicrobial susceptibility testing by GDM was compared with the BMD method in 472 strains (216 *Enterobacteriaceae*, 138 *P. aeruginosa* and 118 *S. aureus*.) and 1652 individual microorganism-antimicrobial agent combinations. There was total agreement between both methods in 98% of combinations. The Kappa index between both techniques was excellent (over 80%). There was only one potential major error for *P. aeruginosa* susceptibility to ceftazidime.

**Conclusions:**

The six GDM strips directly placed on plated bronchial aspirates obtained from patients with a suspicion of VAP provided accurate and reliable susceptibility results within 24 h.

## Introduction

Hospital-acquired pneumonia, and especially ventilator-associated pneumonia (VAP), is one of the leading causes of infection and death in the healthcare setting [1–5]. Its overall attributable mortality has been estimated at 13% [6]. Its incorrect or delayed treatment in the first few hours gives rise to a worse prognosis and higher mortality rate [7–11].

Traditional quantitative cultures of lower respiratory tract (LRT) samples, entailing the isolation, identification, and determination of antimicrobial susceptibility of the pathogens involved usually take 48–72 h. There is therefore a need for rapid diagnostic methods [12]. So far attempts to speed up results have been based on direct antibiotic susceptibility testing of clinical specimens using techniques such as the gradient diffusion susceptibility testing method (GDM) [13]. GDM is an inoculum-tolerant system of proven reliability to rapidly assess the susceptibility of microorganisms directly on positive blood cultures and LRT samples [13–17]. In a previous study, we observed that reporting a rapid GDM result for LRT samples gave rise to fewer days of fever and antibiotic administration until resolution of the VAP episode, reduced antibiotic consumption, less *Clostridium difficile*-associated diarrhoea episodes, lower costs of antimicrobial agents, and fewer days on mechanical ventilation [13].

To date, only susceptibility to traditional antimicrobials has been tested by direct GDM. However, several new drugs have been developed for multiple drug resistant (MDR) pathogens. As far as we know, no study has yet examined the efficacy of direct GDM for testing susceptibility to these new drugs. The present study compares results for susceptibility to ceftazidime, ceftobiprole, ceftolozane-tazobactam, meropenem, doripenem and tedizolid using the rapid direct GDM procedure versus the standard broth microdilution (BMD) method in clinical samples.

## Methods

### Aim, design and setting

#### Study period and clinical samples

Our institution is a 1550-bed university hospital attending a population of approximately 715,000 in Madrid, Spain. The hospital has three different ICUs for adult patients (medical ICU, general postsurgical ICU, and cardiac surgery ICU) with a total of 42 beds.

From September 2015 to November 2016, LRT samples (bronchial aspirates) from intensive care unit (ICU) patients with suspicion of LRT infection acquired during mechanical ventilation were assessed for inclusion in this study. After a Gram stain, samples of suitable quality, as defined by less than ten squamous epithelial cells/low power field and the presence of microorganisms [18, 19], were selected when a predominant morphotype was seen, either Gram-negative bacilli or Gram-positive cocci in clusters. Selected samples were processed for direct GDM antibody susceptibility testing.

#### Processing of samples for direct GDM antibody susceptibility testing

Selected samples were directly spread with a swab (approximately 0.1 mL of sample) onto the surface of a Mueller–Hinton agar plate (15-cm diameter). Six GDM strips (ceftazidime, ceftobiprole, ceftolozane-tazobactam, meropenem, doripenem, and tedizolid) were placed directly onto the plates which were then incubated at 35 °*C. minimum* inhibitory concentration (MIC) readings were performed at 18–24 h under transmitted light. The ceftolozane-tazobactam and tedizolid GDM strips were obtained from MSD, Spain. The remaining strips were from Liofilchem® (Roseto Degli Abruzzi, Italy). To assess the accuracy of the strips *Staphylococcus aureus* ATCC 29213 and *Pseudomonas aeruginosa* ATCC 27853 were used as controls.

#### Standard quantitative culture

All samples were also processed for standard quantitative culture by plating using a calibrated loop (2.5 μL) onto Columbia agar containing 5% sheep blood, colistin-nalidixic acid agar with 5% sheep blood, chocolate agar, and MacConkey agar plates [20]. After 24–48 h of incubation, colonies were counted. Colony counts of ≥10^4^ colony forming units –CFU−/mL of primary pathogens were considered significant, whereas counts below 10^4^ CFU/mL were discarded as negative [20]. The investigator performing the Gram stain was blinded to the culture results of the samples.

Microorganisms were identified by MALDI-TOF MS (Bruker Daltonics, Bremen, Germany) and susceptibility testing performed by BMD using a customized Sensititre® panel (Thermo Fisher Scientific, MA, U.S.) containing ceftazidime, ceftolozane-tazobactam, meropenem and doripenem (Trek Diagnostic Systems, Thermo Scientific, Ohio, U.S.). Ceftolozane-tazobactam susceptibility was tested using a fixed concentration of 4 mg/L of tazobactam. Minimum inhibitory concentrations (MICs) for ceftobiprole and tedizolid were determined by BMD, as indicated by the Clinical and Laboratory Standards Institute (CLSI) [21, 22]. Breakpoints were determined according to the CLSI guidelines. For purposes of comparison between the direct GDM and BMD method, ceftobiprole and tedizolid were assessed for Gram-positive microorganisms, and ceftazidime, ceftolozane-tazobactam, doripenem and meropenem for Gram-negative microorganisms. *Staphylococcus aureus* ATCC 29213 and *Pseudomonas aeruginosa* ATCC 27853 were used as controls.

#### Definitions and interpretation of results

Individual organism–antimicrobial agent comparisons were made between the direct GDM and BMD tests. The results obtained were recorded as follows: “total agreement”, when the MICs obtained using the GDM and BMD were identical or differed by only one two-fold dilution; “very major error”, when the MIC obtained by GDM classified the microorganism as susceptible, while the MIC obtained by BMD classified it as resistant; “major error”, when the MIC obtained by GDM classified the microorganism as resistant and that obtained by BMD as susceptible; and “minor error”, when the MIC obtained by GDM classified the microorganism as showing intermediate susceptibility and that obtained by BMD as susceptible or resistant and vice-versa.

Percentiles 50 and 90 were calculated for the results’ distributions. After categorising the numerical GDM and BMD results into susceptible/intermediate/resistant, correlations were calculated through the Kappa index (IBM® SPSS®, ver. 15.0.).

## Results

**Fig. 1:**
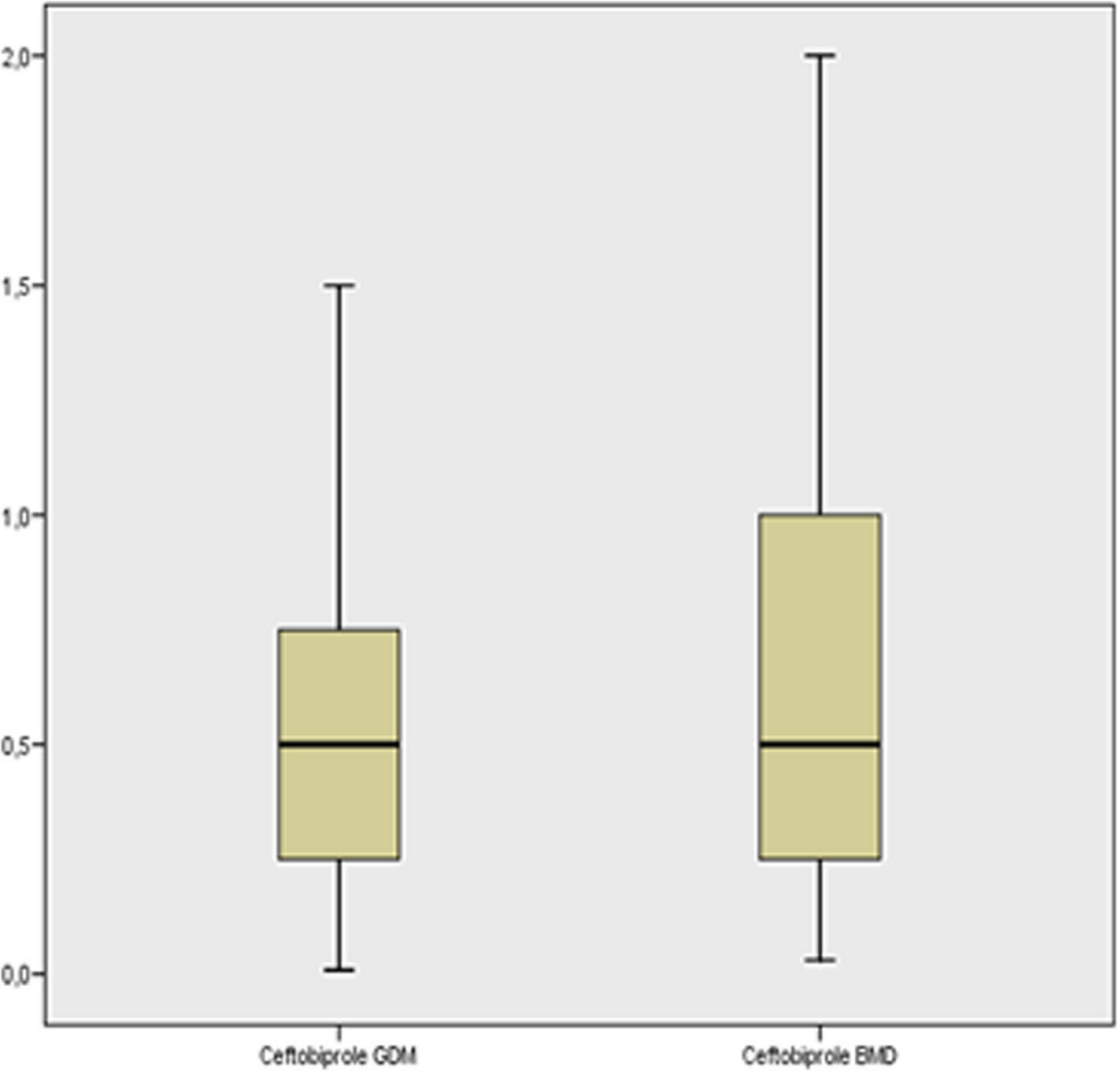
Box plot of minimum inhibitory concentrations (mg/dL) for ceftobiprole using the two tests. GDM = gradient diffusion method, BMD = broth microdilution method

**Fig. 2:**
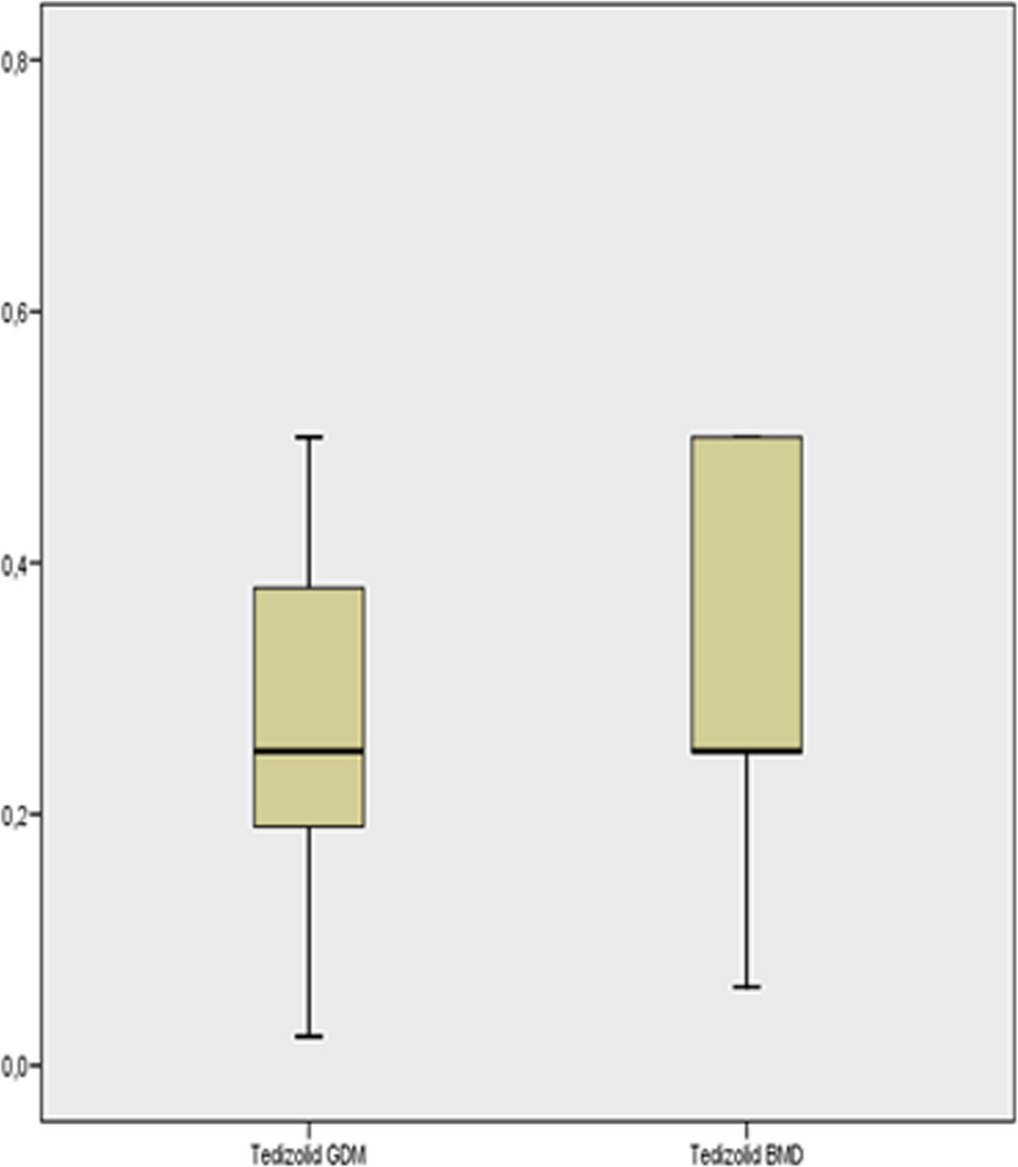
Box plot of minimum inhibitory concentrations (mg/dL) for tedizolid using the two tests. GDM: gradient diffusion method. BMD: broth microdilution method

Over the study period, we received 1376 bronchial aspirates from 672 adult patients. After Gram staining, we selected 445 samples fulfilling the inclusion criteria (good quality microbiological samples with a predominant single morphotype). By direct plating on Mueller-Hinton agar, we recovered 504 (95.5%) of the 528 microorganisms retrieved by the standard semiquantitative method. GDM and BMD antimicrobial susceptibility tests were compared in 472 strains (216 *Enterobacteriaceae*, 138 *P. aeruginosa* and 118 *S. aureus*) and 1652 individual microorganism-antimicrobial agent combinations. Results are presented in Tables 1, 2, 3, and Figs. 1 and 2. There was total agreement between both methods in 98% of the combinations. The Kappa index between both techniques was excellent (over 80%). There was only one very major error (an isolate of *P. aeruginosa* classified as susceptible to ceftazidime by GDM and yet confirmed resistant by BMD).

**Table 1:** *Enterobacteriaceae* (216 strains): results of GDM versus BMD as the reference method

Antibiotic/method	Range	MIC 50	MIC 90	Susceptible (%)	Intermediate (%)	Resistant (%)	Kappa	Minor error (%)	Major and very major error (%)
Ceftazidime GDM	0.016- > 256	0.25	32	186 (86.1)	6 (2.8)	24 (11.1)	88.8	5 (2.3)	1 (0.5)
Ceftazidime BMD	< 0.06- > 32	0.25	32	185 (85.6)	5 (2.3)	26 (12.0)	–	–	–
Ceftolozane-tazobactam GDM	0.023- > 256	0.25	1.5	197 (91.2)	6 (2.8)	13 (6.0)	91.1	3 (1.4)	–
Ceftolozane-tazobactam BMD	< 0.06- > 32	0.25	1	199 (92.1)	5 (2.3)	12 (5.6)	–	–	–
Meropenem GDM	< 0.002- > 32	0.032	0.125	209 (96.8)	1 (0.5)	6 (2.8)	92.6	1 (0.5)	–
Meropenem BMD	< 0.06- > 32	< 0.06	1	209 (96.8)	–	7 (3.2)	–	–	–
Doripenem GDM	< 0.002- > 32	0.064	0.25	209 (96.8)	–	7 (3.2)	100	–	–
Doripenem BMD	< 0.06- > 32	< 0.06	0.5	209 (96.8)	–	7 (3.2)	–	–	–

Range, MIC 50 and MIC 90 are given in mg/mL. GDM = gradient diffusion method, BMD = broth microdilution method

**Table 2:** *Pseudomonas aeruginosa* (138 strains): results of GDM versus BMD as the reference method

Antibiotic/method	Range	MIC 50	MIC 90	Susceptible (%)	Intermediate (%)	Resistant (%)	Kappa	Minor error (%)	Major and very major error (%)
Ceftazidime GDM	0.064- > 256	2	192	102 (73.9)	13 (9.4)	23 (16.7)	81.7	9 (6.5)	1 (0.7)*
Ceftazidime BMD	< 0.06- > 32	4	> 32	106 (76.8)	6 (4.3)	26 (18.8)	–	–	–
Ceftolozane-tazobactam GDM	0.047- > 256	0.5	1.5	135 (97.8)	–	3 (2.2)	85.4	1 (0.7)	–
Ceftolozane-tazobactam BMD	< 0.06- > 64	0.5	4	134 (97.1)	1 (0.7)	3 (2.2)	–	–	–
Meropenem GDM	0.023- > 32	4	> 32	62 (44.9)	20 (14.5)	56 (40.6)	92.9	6 (4.3)	–
Meropenem BMD	< 0.06- > 32	4	> 32	63 (45.7)	20 (14.5)	55 (39.9)	–	–	–
Doripenem GDM	0.004- > 32	4	> 32	60 (43.5)	25 (18.1)	53 (38.4)	89.6	8 (5.8)	1 (0.7)
Doripenem BMD	< 0.06- > 32	4	32	62 (44.9)	25 (18.1)	51 (37.0)	–	–	–

Range, MIC 50 and MIC 90 are given in mg/mL. GDM = gradient diffusion method, BMD = broth microdilution method. *This was a very major error i.e., susceptible by GDM and resistant by BMD

**Table 3:** *Staphylococcus aureus* (118 strains): results of GDM versus BMD as the reference method

Antibiotic/method	Range	MIC 50	MIC 90	Susceptible (n,%)	Intermediate (n,%)	Resistant (n,%)	Kappa	Minor error	Major and very mayor error
Ceftobiprole GDM	0.008–2	0.5	1.5	118 (100)	0 (0)	0 (0)	100	–	–
Ceftobiprole BMD	0.03–2	0.5	2	118 (100)	0 (0)	0 (0)	–	–	–
Tedizolid GDM	0.023–0.75	0.19	0.5	115 (97.5)	3 (2.5)	0 (0)	97.5	3 (2.5)	0 (0)
Tedizolid BMD	0.0625–0.5	0.25	0.5	118 (100)	0 (0)	0 (0)	–	–	–

Range, MIC 50 and MIC 90 are given in mg/mL. GDM = gradient diffusion method, BMD = broth microdilution method, MIC = minimum inhibitory concentration

*Staphylococcus aureus* grew in 118 of the samples: in 80 samples they were methicillin-susceptible (MSSA) strains and in 38 they were methicillin-resistant (MRSA). These data are provided in Table 3. All strains were susceptible to both ceftobiprole and tedizolid. Three samples returned an intermediate result by direct GDM tedizolid susceptibility testing and were classified as susceptible by BMD. This could be explained by an inoculum effect (the concentration of microorganisms in the clinical samples was higher than in the inoculum used in the reference method). Correlation was excellent (Kappa index 100%).

## Discussion

The results of our study indicate that, in patients with suspicion of VAP, direct GDM testing of susceptibility to new antibiotics provides accurate results on the day after sample processing, compared to the standard method, which takes longer than 48–72 h.

Sample selection for this study was by Gram staining. This procedure used on lower respiratory tract samples (sputum, bronchial aspirates, broncho-alveolar lavage) has proven useful for the etiological diagnosis of both community-acquired and hospital-acquired pneumonia [23–29]. In effect, the Gram stain result is part of the Clinical Pulmonary Infection Score (CPIS) score for VAP diagnosis [30]. Because of its high negative predictive capacity [31], a negative Gram stain result will rule out significant bacterial counts in broncho-alveolar lavage samples with a high degree of certainty (97.6%). Such a negative result may thus allow for the use of narrow-spectrum antibiotics or withholding empiric antimicrobial therapy in patients with suspicion of VAP [24].

The direct antibiotic susceptibility testing method (GDM) offers preliminary information for the rapid prescription of adequate antibiotic treatment [16]. Indeed, this method may have a significant impact on the best choice of antibiotic therapy (more effective or lower-priced drugs) and leads to reduced mortality, a lower number of additional laboratory and radiology diagnostic tests, and shorter ICU stay [17, 32]. In the context of VAP, it helps clinicians administer a targeted antibiotic therapy in under 24 h after clinical suspicion of pneumonia. Rapid information for physicians based on the direct GDM has been also associated with less misappropriate use of antibiotics, fewer days on mechanical ventilation, fewer *C. difficile* associated diarrhoea episodes, less adverse events related to antimicrobials, and reduced costs [13].

Until the present study, GDM had not been validated for testing susceptibility to the newer antibiotics. Our findings indicate 98% total agreement for the susceptibility of individual microorganism-antimicrobial agent combinations, and only 24 (4.5) of the 528 microorganisms recovered by the standard semiquantitative method were missed. These microorganisms not picked up by the method were strains of pathogens unable to grow on Mueller-Hinton agar, such as *Streptococcus pneumoniae*, *Haemophilus* spp. or *Moraxella catarrhalis,* which were, nevertheless*,* recovered in the culture media employed for the semiquantitative reference method determining that information about the existence of these microorganisms was not lost. When in the Gram stain characteristic Gram-positive diplococci are observed, in our laboratory we add a plate of Columbia agar with 5% sheep’s blood on which a cefotaxime GDM strip is placed so that we know the susceptibility of *S. pneumoniae* to this antibiotic as soon as possible.

The major errors observed in our study (microorganisms classified as resistant by GDM yet sensitive by BMD) are likely explained by the inoculum effect. This effect is particularly observed for betalactam antibiotics in which it is easier to obtain a classification of resistant by the direct method if the inoculum is large. We are unable to offer an explanation for the very major error obtained here for the susceptibility of *P. aeruginosa* to ceftazidime.

Reading of the GDM method is easy even with polymicrobial cultures in which different microorganisms with different levels of resistance can be found. The method also allows direct reading of MIC inhibiting all the morphotypes in a sample [33]. Further, resistant microorganisms that grow inside the ellipse can be determined and isolated, and then coupled with MALDI-TOF MS to identify all morphotypes. This method has also been used successfully in patients with cystic fibrosis, in which it enables selection of the best targeted antibiotic treatment [34].

The antibiotics tested here are those active against the microorganisms that most often cause VAP such as MRSA (19.5–32%), *P. aeruginosa* (21–27%), *Enterobacter* spp. (7–9%), *Klebsiella* spp. (7–10%) and *Acinetobacter* spp. (5–14%) [35]. For instance, carbapenems, are widely used in ICUs, given the high frequency of multi-resistant Gram-negative bacilli. Further, the ceftolozane-tazobactam, combination covers ESBL-producing *Enterobacteriaceae* and carbapenem-resistant *P. aeruginosa*, with the most common resistance mechanisms in this microorganism (up-regulated efflux and derepressed AmpC) [36]. The antibiotics selected against Gram-positives were ceftobiprole and tedizolid, both active against MRSA, with excellent intrinsic activity [37, 38]. We have just closed a randomized, double-blind, phase III study, comparing intravenous 200 mg of tedizolid for seven days or linezolid for ten days for the treatment of suspected or confirmed hospital-acquired pneumonia requiring intubation or VAP.

Among the limitations of this study, we could mention that because bronchial aspirate samples are sometimes very viscous it is difficult to spread them evenly over the surface of the agar. We would therefore recommend liquefying the sample by adding a mucolytic agent, such as dithiothreitol, and then adequately vortexing and mixing them [39]. Another limitation is that only six antibodies may be tested at a time, as more GDM strips will not fit on the Mueller-Hinton agar plate. Finally, this was a single centre study and its results might not be extrapolatable to other centres with different populations.

## Conclusions

Ceftazidime, ceftolozane-tazobactam, linezolid, meropenem, doripenem and tedizolid GDM strips placed directly on plated bronchial aspirates preselected from patients with suspicion of VAP provided accurate and reliable results within 24 h. In 98% of cases there was total agreement between this method and the broth microdilution method. Further, the Kappa index between both techniques was excellent (over 80%). Although larger multicentre studies are needed, this approach could have a significant impact on antibiotic stewardship in intensive care units.

## Data Availability

The datasets used and/or analysed durgin the current study are available from the corresponding author on reasonable request.

## Abbreviations

AmpC: ampicillin-resistance gene group C betalactamase
ATCC: American type culture collection
BMD: broth microdilution method
CFU: colony forming units
CLSI: clinical and Laboratory Standards Institute
CPIS: clinical pulmonary infection score
ESBL: extended spectrum beta lactamase
GDM: gradient diffusion susceptibility testing method
ICU: intensive care unit
LRT: lower respiratory tract
MALDI-TOF MS: matrix-assisted laser desorption/ionization time-of-flight mass spectrometry
MDR: multiple drug resistant
MIC: minimum inhibitory concentration
MRSA: methicillin-resistant *Staphylococcus aureus*
MSD: Merck Sharp & Dohme
MSSA: methicillin-susceptible *Staphylococcus aureus*
VAP: ventilator-associated pneumonia

## References

[1] Hortal J, Munoz P, Cuerpo G, Litvan H, Rosseel PM, Bouza E (2009). Ventilator-associated pneumonia in patients undergoing major heart surgery: an incidence study in Europe. Crit Care.

[2] Tamayo E, Alvarez FJ, Martinez-Rafael B, Bustamante J, Bermejo-Martin JF, Fierro I, Eiros JM, Castrodeza J, Heredia M, Gomez-Herreras JI (2012). Ventilator-associated pneumonia is an important risk factor for mortality after major cardiac surgery. J Crit Care.

[3] Giunta V, Ferrer M, Esperatti M, Ranzani OT, Saucedo LM, Li Bassi G, Blasi F, Torres A (2013). ICU-acquired pneumonia with or without etiologic diagnosis: a comparison of outcomes. Crit Care Med.

[4] He S, Chen B, Li W, Yan J, Chen L, Wang X, Xiao Y (2014). Ventilator-associated pneumonia after cardiac surgery: a meta-analysis and systematic review. J Thorac Cardiovasc Surg.

[5] Corrado RE, Lee D, Lucero DE, Varma JK, Vora NM (2017). Burden of adult community-acquired, health-care-associated, hospital-acquired, and ventilator-associated pneumonia: New York City, 2010 to 2014. Chest..

[6] Ferrer M, Torres A (2018). Epidemiology of ICU-acquired pneumonia. Curr Opin Crit Care.

[7] Siempos II, Vardakas KZ, Kyriakopoulos CE, Ntaidou TK, Falagas ME (2010). Predictors of mortality in adult patients with ventilator-associated pneumonia: a meta-analysis. Shock..

[8] Kett DH, Cano E, Quartin AA, Mangino JE, Zervos MJ, Peyrani P, Cely CM, Ford KD, Scerpella EG, Ramirez JA (2011). Implementation of guidelines for management of possible multidrug-resistant pneumonia in intensive care: an observational, multicentre cohort study. Lancet Infect Dis.

[9] Muscedere JG, Shorr AF, Jiang X, Day A, Heyland DK, Canadian Critical Care Trials G (2012). The adequacy of timely empiric antibiotic therapy for ventilator-associated pneumonia: an important determinant of outcome. J Crit Care.

[10] Piskin N, Aydemir H, Oztoprak N, Akduman D, Comert F, Kokturk F, Celebi G (2012). Inadequate treatment of ventilator-associated and hospital-acquired pneumonia: risk factors and impact on outcomes. BMC Infect Dis.

[11] Koulenti D, Tsigou E, Rello J (2017). Nosocomial pneumonia in 27 ICUs in Europe: perspectives from the EU-VAP/CAP study. Eur J Clin Microbiol Infect Dis.

[12] Kollef MH (2007). Moving towards real-time antimicrobial management of ventilator-associated pneumonia. Clin Infect Dis.

[13] Bouza E, Torres MV, Radice C, Cercenado E, de Diego R, Sanchez-Carrillo C, Munoz P (2007). Direct E-test (AB biodisk) of respiratory samples improves antimicrobial use in ventilator-associated pneumonia. Clin Infect Dis.

[14] Bolmström A, Arvidson S, Ericsson M, Karlsson A. A novel technique for direct quantification of antimicrobial susceptibility of microorganisms. Poster 1209. 28th Interscience Conference on Antimicrobial Agents and Chemotherapy; 1988; Los Angeles.

[15] Baker CN, Stocker SA, Culver DH, Thornsberry C (1991). Comparison of the E test to agar dilution, broth microdilution, and agar diffusion susceptibility testing techniques by using a special challenge set of bacteria. J Clin Microbiol.

[16] Kontopidou F, Galani I, Panagea T, Antoniadou A, Souli M, Paramythiotou E, Koukos G, Karadani I, Armaganidis A, Giamarellou H (2011). Comparison of direct antimicrobial susceptibility testing methods for rapid analysis of bronchial secretion samples in ventilator-associated pneumonia. Int J Antimicrob Agents.

[17] Boyer A, Medrano J, Mzali F, Balick-Weber CC, Bessede E, Picard W, Clouzeau B, Bebear CM, Vargas F, Hilbert G (2012). Direct testing of bronchoalveolar lavages from ventilator-associated pneumonia patients. Diagn Microbiol Infect Dis.

[18] Cercenado E, Cercenado S, Marin M, Rico MV, Vicente T, Bouza E (2007). Evaluation of direct E-test on lower respiratory tract samples: a rapid and accurate procedure for antimicrobial susceptibility testing. Diagn Microbiol Infect Dis.

[19] Chan WW: Gram stain. Rejection criteria for sputum and endotracheal aspirates for culture. In: Leber AL, editor. Clinical Microbiology Procedures Handbook*.* Washington, DC: ASM Press; 2016. p. 3.2.1.20.

[20] Gilligan PH, Alby K, York MK: Lower respiratory tract cultures. In: American Society for Microbiology, editor. Clinical Microbiology Procedures Handbook*.* Washington, D.C.: ASM Press; 2016. p.3.11.12.11–17.

[21] Clinical and Laboratory Standards Institute (CLSI). Methods for dilution antimicrobial susceptibility tests for bacteria that grow aerobically: approved standard. 10th ed. CLSI document M07-A10 (ISBN 1–56238–987-4 [Print]; ISBN 1–56238–988-2 [Electronic]). Clinical and Laboratory Standards Institute, 950 West Valley Road, Suite 2500, Wayne, Pennsylvania 19087 USA, 2015.

[22] Clinical and Laboratory Standards Institute (CLSI). Performance Standards for Antimicrobial Susceptibility Testing: Twenty-Fifth Infomation Supplement. CLSI document M100-S25(ISBN 1–56238–989-0 [Print]; ISBN 1–56238–990-4 [Electronic]). Clinical and Laboratory Standards Institute, 950 West Valley Road, Suite 2500, Wayne, Pennsylvania 19087 USA, 2015.

[23] Marquette CH, Georges H, Wallet F, Ramon P, Saulnier F, Neviere R, Mathieu D, Rime A, Tonnel AB (1993). Diagnostic efficiency of endotracheal aspirates with quantitative bacterial cultures in intubated patients with suspected pneumonia. Comparison with the protected specimen brush [see comments]. Am Rev Respir Dis.

[24] Laupland KB, Church DL, Gregson DB (2005). Validation of a rapid diagnostic strategy for determination of significant bacterial counts in bronchoalveolar lavage samples. Arch Pathol Lab Med.

[25] Matsushima A, Tasaki O, Shimizu K, Tomono K, Ogura H, Shimazu T, Sugimoto H (2008). Preemptive antibiotic treatment based on gram staining reduced the incidence of ARDS in mechanically ventilated patients. J Trauma.

[26] Miyashita N, Shimizu H, Ouchi K, Kawasaki K, Kawai Y, Obase Y, Kobashi Y, Oka M (2008). Assessment of the usefulness of sputum gram stain and culture for diagnosis of community-acquired pneumonia requiring hospitalization. Med Sci Monit.

[27] Anevlavis S, Petroglou N, Tzavaras A, Maltezos E, Pneumatikos I, Froudarakis M, Anevlavis E, Bouros D (2009). A prospective study of the diagnostic utility of sputum gram stain in pneumonia. J Inf Secur.

[28] Jung B, Embriaco N, Roux F, Forel JM, Demory D, Allardet-Servent J, Jaber S, La Scola B, Papazian L (2010). Microbiogical data, but not procalcitonin improve the accuracy of the clinical pulmonary infection score. Intensive Care Med.

[29] Fukuyama H, Yamashiro S, Kinjo K, Tamaki H, Kishaba T (2014). Validation of sputum gram stain for treatment of community-acquired pneumonia and healthcare-associated pneumonia: a prospective observational study. BMC Infect Dis.

[30] Pugin J, Auckenthaler R, Mili N, Janssens JP, Lew PD, Suter PM (1991). Diagnosis of ventilator-associated pneumonia by bacteriologic analysis of bronchoscopic and nonbronchoscopic "blind" bronchoalveolar lavage fluid. Am Rev Respir Dis.

[31] Singh N, Rogers P, Atwood CW, Wagener MM, Yu VL (2000). Short-course empiric antibiotic therapy for patients with pulmonary infiltrates in the intensive care unit. A proposed solution for indiscriminate antibiotic prescription. Am J Respir Crit Care Med.

[32] Doern GV, Vautour R, Gaudet M, Levy B (1994). Clinical impact of rapid in vitro susceptibility testing and bacterial identification. J Clin Microbiol.

[33] Zebouh M, Thomas C, Honderlick P, Lemee L, Segonds C, Wallet F, Husson MO (2008). Direct antimicrobial susceptibility testing method for analysis of sputum collected from patients with cystic fibrosis. J Cyst Fibros.

[34] Zebouh M, Thomas C, Honderlick P, Lemee L, Segonds C, Wallet F, Husson MO (2005). Evaluation of a new E-test method for antimicrobial sensitivity testing of *Pseudomonas aeruginosa* isolates from cystic fibrosis. Pathol Biol (Paris).

[35] Farrell DJ, Castanheira M, Mendes RE, Sader HS, Jones RN (2012). In vitro activity of ceftaroline against multidrug-resistant *Staphylococcus aureus* and *Streptococcus pneumoniae*: a review of published studies and the AWARE surveillance program (2008-2010). Clin Infect Dis.

[36] Livermore DM, Mushtaq S, Meunier D, Hopkins KL, Hill R, Adkin R, Chaudhry A, Pike R, Staves P, Woodford N (2017). Activity of ceftolozane/tazobactam against surveillance and 'problem' *Enterobacteriaceae*, *Pseudomonas aeruginosa* and non-fermenters from the British Isles. J Antimicrob Chemother.

[37] Farrell DJ, Flamm RK, Sader HS, Jones RN (2014). Ceftobiprole activity against over 60,000 clinical bacterial pathogens isolated in Europe, Turkey, and Israel from 2005 to 2010. Antimicrob Agents Chemother.

[38] Bassetti M, Vena A, Castaldo N, Righi E, Peghin M (2018). New antibiotics for ventilator-associated pneumonia. Curr Opin Infect Dis.

[39] Hirsch SR, Zastrow JE, Kory RC (1969). Sputum liquefying agents: a comparative in vitro evaluation. J Lab Clin Med.

